# Knockdown NRPC2, 3, 8, NRPABC1 and NRPABC2 Affects RNAPIII Activity and Disrupts Seed Development in Arabidopsis

**DOI:** 10.3390/ijms222111314

**Published:** 2021-10-20

**Authors:** Hailiang Zhao, Yao Qin, Ziyi Xiao, Kun Liang, Dianming Gong, Qin Sun, Fazhan Qiu

**Affiliations:** National Key Laboratory of Crop Genetic Improvement, Hubei Hongshan Laboratory, College of Plant Science and Technology, Huazhong Agricultural University, Wuhan 430070, China; hailiang@webmail.hzau.edu.cn (H.Z.); yaoqin@webmail.hzau.edu.cn (Y.Q.); xiaoziyi_827@webmail.hzau.edu.cn (Z.X.); liangkun@webmail.hzau.edu.cn (K.L.); gongdianming@mail.hzau.edu.cn (D.G.); qinsun@webmail.hzau.edu.cn (Q.S.)

**Keywords:** RNA polymerase III subunits, 5S rRNA, tRNA, seed development, Arabidopsis

## Abstract

RNA polymerase III (RNAPIII) contains 17 subunits forming 4 functional domains that control the different stages of RNAPIII transcription and are dedicated to the synthesis of small RNAs such as 5S rRNA and tRNAs. Here, we identified 23 genes encoding these subunits in Arabidopsis (*Arabidopsis thaliana*) and further analyzed 5 subunits (NRPC2, NRPC3, NRPC8, NRPABC1, and NRPABC2) encoded by 6 genes with different expression patterns and belonging to different sub-complexes. The knockdown of these genes repressed the expression of 5S rRNA and tRNAs, causing seed developmental arrest at different stages. Among these knockdown mutants, RNA-seq analysis revealed 821 common differentially expressed genes (DEGs), significantly enriched in response to stress, abscisic acid, cytokinins, and the jasmonic acid signaling pathway. Weighted gene co-expression network analysis (WGCNA) revealed several hub genes involved in embryo development, carbohydrate metabolic and lipid metabolic processes. We identified numerous unique DEGs between the mutants belonging to pathways, including cell proliferation, ribosome biogenesis, cell death, and tRNA metabolic processes. Thus, *NRPC2*, *NRPC3*, *NRPC8*, *NRPABC1*, and *NRPABC2* control seed development in Arabidopsis by influencing RNAPIII activity and, thus, hormone signaling. Reduced expression of these subunit genes causes an insufficient accumulation of the total RNAPIII, leading to the phenotypes observed following the genetic knockdown of these subunits.

## 1. Introduction

In Arabidopsis (*Arabidopsis thaliana*), seed development starts with a double fertilization event that is followed by rapid proliferation and the expansion of the endosperm. It ends when embryo growth takes place at the expense of the endosperm. This process is controlled by several signaling pathways, including the G-protein signaling, the IKU pathway, the mitogen-activated protein kinase signaling pathway, the ubiquitin–proteasome pathway, and the pathways involving phytohormones and transcriptional regulatory factors [1,2,3]. Identifying these regulatory pathways, and conducting functional analyses of their regulators, has greatly expanded our knowledge of seed development. However, there remains a need for more information regarding the control of seed development in Arabidopsis.

RNAPIII is the largest of the three eukaryotic RNA polymerases. Its holoenzyme comprises 17 subunits, organized into 4 functional domains: the enzymatic core, the heterotrimeric subcomplex, the heterodimeric subcomplex, and a two-subunit peripheral stalk [4,5]. The enzymatic core is structurally conserved across eukaryotic RNA polymerases and contains ten subunits: NRPC1, NRPC2, NRPABC1, NRPABC2, NRPABC3, NRPABC4, NRPABC5, NRPAC1, NRPAC2, and NRPC10 [6]; RNAPIII subunits 1 (NRPC1) and 2 (NRPC2) both interact with the other subunits to form the active domain of RNAPIII [4]. The heterotrimeric subcomplex comprises the subunits NRPC3, NRPC6, and NRPC7 [7], which are involved in the recruitment of other RNAPIII subunits to TFIIIB [8], and the initiation of transcription [9,10,11]. The NRPC4–NRPC5 heterodimer participates in transcriptional initiation and termination [12,13,14]. The peripheral stalk comprises NRPC8 and NRPC9 and is involved in the initiation of transcription [6], and binding to single-stranded RNA [15]. Although the molecular structures and transcription mechanisms of RNAPIII have been identified in the yeast *accharomyces cerevisiae*, many of the components in these complexes have not yet been functionally characterized in plants.

Several mutant RNAPIII subunits have been reported in previous studies. RNAPIII-related leukodystrophy, caused by variation in NRPC1, NRPC2, NRPC10, and NRPAC1, often impairs the normal assembly, or biogenesis, of RNAPIII and causes a retention of the unassembled subunits in the cytoplasm [16,17]. A rare homozygous mutation (D40H) in NRPC5 impairs the assembly of RNAPIII initiation complexes and causes an innate immune deficiency state in humans [18]. In yeast, the mutation of NRPC4 preferentially arrests cell division in the G1 phase and results in large, round, unbudded, and temperature-sensitive cells [19]. Furthermore, a splice-site substitution of NRPC2 suppresses RNAPIII transcriptional activity, disrupting the proliferation and growth of tissue progenitor cells in the *zebrafish* digestive system [20]. The mutation of NRPC2 in mice reduces intestinal epithelial cell proliferation and impaired crypt development [21]; meanwhile, loss of NRPC2 function represses cell proliferation and endoreduplication in maize endosperm [22]. Despite these findings, we noticed that these mutants only occupy small parts of all subunits of RNAPIII, indicating the difficulty of isolating mutations in other RNAPIII subunits. Therefore, creating new mutants in RNAPIII subunits using different biotechnologies is still warranted to provide further understanding of the components and transcription machinery of RNAPIII.

In this study, we identified 17 subunits of RNAPIII in Arabidopsis. We functionally characterized five of these (NRPC2, NRPC3, NRPC8, NRPABC1, and NRPABC2), which had different expression patterns and belonged to different sub-complexes. Knocking down the genes encoding these subunits caused reduced expression of in 5S rRNA and 16 tRNAs, arresting seed development at the globular stage, early cotyledon stage, pre-globular stage, early cotyledon stage and early cotyledon stage in *nrpc2*, *nrpc3*, *nrpc8*, *nrpabc1*, and *nrpabc2* mutants, respectively. Further analysis indicated that the process of response to stress, and hormones such as abscisic acid (ABA) and jasmonic acid (JA), is altered when the genes encoding these subunits are knocked down. Meanwhile, we identified several unique differentially expressed genes (DEGs) in seeds with different mutant subunits belonging to distinct pathways. Thus, our work has identified the subunits of RNAPIII in Arabidopsis, functionally characterized five representative subunits of RNAPIII, and demonstrated that these subunits control seed development by regulating RNAPIII-mediated transcription.

## 2. Results

### 2.1. Subunits of RNAPIII Are Encoded by 23 Genes in Arabidopsis

To characterize the subunits of RNAPIII in Arabidopsis, we searched the non-redundant protein sequence database for Arabidopsis by querying the amino acid sequences of RNAPIII subunits in *S. cerevisiae* and humans (*Homo sapiens*). Then, we redressed our results according to the mass spectrometry of RNAPIII subunits determined by Ream et al. [23]. From this database, we identified 23 genes encoding the 17 subunits of RNAPIII in Arabidopsis (Table 1). Six of the 17 subunits (NRPC4, NRPC7, NRPC9, NRPC10, NRPABC1, and NRPABC2) were encoded by 2 paralogs, and 11 subunits were encoded by unique genes (Table 1).

### 2.2. RNAPIII Subunits Have Different Expression Patterns during Seed Development

**Figure 1:**
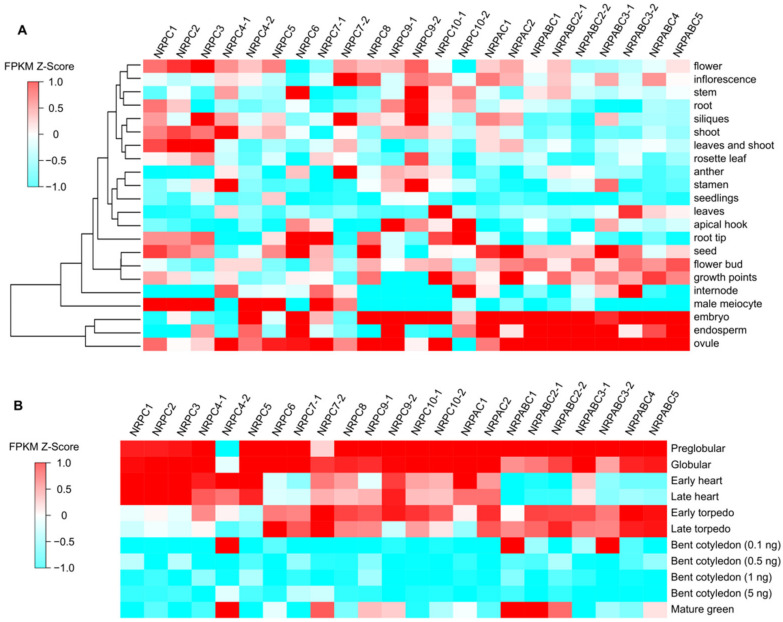
Expression patterns of RNAPIII subunits in Arabidopsis. (**A**) Expression patterns in different tissues and organs. (**B**) Expression patterns in seeds at different stages of development.

The expression patterns of different RNAPIII subunits in Arabidopsis were investigated by analyzing the transcripts using publicly available transcriptome sequencing (RNA-seq) data [24]. The subunits genes, *NRPABC1*, *NRPABC2*, *NRPABC3*, *NRPABC4,* and *NRPABC5,* exhibited high-level expression in different seed tissues, including embryos, endosperm and ovules at the pre-globular, globular, early torpedo, and late torpedo stages, whereas *NRPABC1* and *NRPABC2* had a higher expression level in the mature green seed (Figure 1). Subunits genes *NRPAC1* and *NRPAC2* were expressed in seeds, and their expression levels steadily decreased advancing seed growth. *NRPC1*, *NRPC2,* and *NRPC3* were highly expressed in the male meiocyte, flower, leaf and shoot, and those subunits were highly expressed at the early development stage of seed development (Figure 1). NRPC4 was encoded by two gene paralogs with different expression patterns in different tissues and at different stages of seed development. *NRPC4-1* was highly expressed in the shoot, stamen, and ovule, but *NRPC4-2* was abundantly expressed in male meiocytes and embryos (Figure 1). The expression of *NRPC4-1* decreased steadily with advancing seed growth, whereas *NRPC4-2* was expressed at a high level in the early heart, bent cotyledon (0.1 ng), and mature green seed stages. *NRPC5* was expressed at a high level in male meiocytes and ovules and was detected during the early stages of seed development. *NRPC6* was detected in stem, root tip, and seed tissues, including embryos, endosperm, and ovules, with high expression levels at the pre-globular, globular, and torpedo stages of seed development (Figure 1). *NRPC7-1* and *NRPC7-2* had different expression patterns. *NRPC7-1* was strongly expressed in male meiocytes, root tips, and ovules, while *NRPC7-2* was detected in inflorescence, siliques, and anthers. *NRPC7-1* was expressed at the pre-globular, globular stages; however, the expression level of *NRPC7-2* first increased, and then decreased, during seed development (Figure 1). *NRPC8* was specifically expressed in seed organs, including the embryo and ovule, and the expression level decreased during seed development (Figure 1). *NRPC9-1* was expressed in the apical hook, embryo, endosperm, and ovule, but *NRPC9-2* was expressed more ubiquitously, including in stem, root, siliques, stamen, and embryo tissues. *NRPC9-1* and *NRPC9-2* were expressed at the early stages of seed development (Figure 1). *NRPC10-1* exhibited high expression in leaves, growth points, embryos, and ovules, but *NRPC10-2* was detected in the apical hook, root tip, internode, and endosperm; *NRPC10-1* and *NRPC10-2* were expressed early in seed development (Figure 1).

RNAPIII comprises 17 subunits encoded by 23 genes in Arabidopsis. According to their expression patterns during seed development, these genes can be clustered into two categories. The first category comprises nine genes encoding seven subunits (*NRPAC1*, *NRPAC2*, *NRPABC1*, *NRPABC2*, *NRPABC3*, *NRPABC4*, and *NRPABC5*), with high expression levels in the globular and torpedo stages of seed development. This suggests that these subunits are important in cell proliferation and cell differentiation during seed development (Figure 1). The second category included genes encoding the other 10 subunits; these were ubiquitously expressed, but most were detected in embryos or ovules. These genes were expressed in the early stage, and their expression gradually declined during seed development, coinciding with the initiation of seed development (Figure 1).

### 2.3. Knockdown of Five RNAPIII Subunits Leads to Seed Development Arrest at Different Stage in Arabidopsis

**Figure 2:**
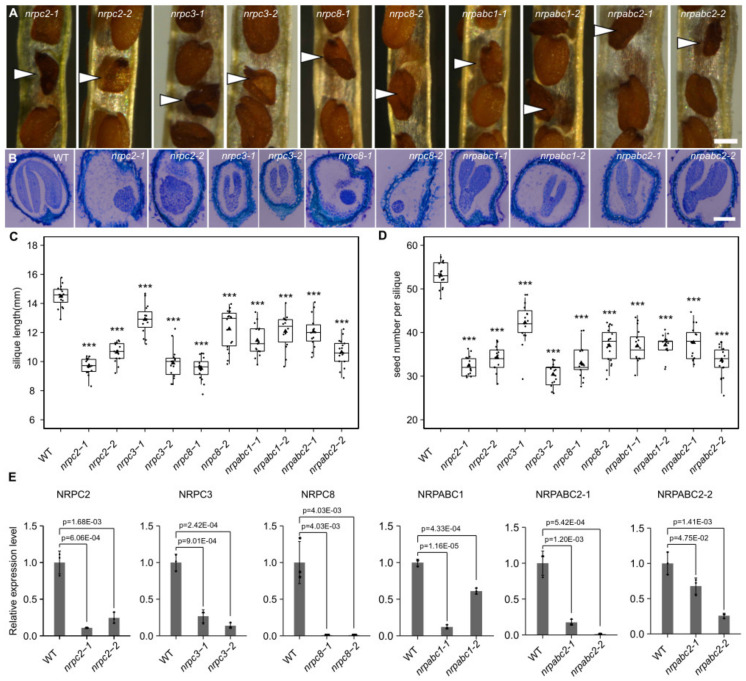
Phenotypic features of seeds with knockdown of different subunits genes, and analysis of the expression of different subunits in seeds of wild-type and different subunit knockdown lines. (**A**) Phenotypic features of mature seeds (white arrows indicate mutant seeds). Scale bar = 300 µm. (**B**) Longitudinal paraffin sections of developing seeds at 15DAF. Scale bar = 100 µm. (**C**) Average silique length at 10 DAF. Asterisks indicate significant difference by Student’s *t*-test (“***” *p* < 0.001). (**D**) Seed number per silique. Asterisks indicate significant difference by Student’s *t*-test (“***” *p* < 0.001). (**E**) RT-qPCR analysis of different subunits-encoding genes in seeds at 10 DAF of wild-type and different RNAPIII subunit knockdown lines. All expression levels are normalized to that of *ACTIN*. Three replicates for each sample were performed, and data are means ± SD. *p*-values determined by Student’s *t*-test.

We selected five subunits belonging to different RNAPIII sub-complexes, showing different expression patterns during Arabidopsis seed development. We induced the antisense RNA of these genes to analyze the function of RNAPIII during seed development and characterized the phenotypes of 10 mutant Arabidopsis lines in which seed development had arrested. Specifically, we found that the relative expression level of *NRPC2* was reduced by 75% and 90% in *nrpc2-2* and *nrpc2-1*, compared with control lines (Figure 2E); the mutant embryos developed more slowly than the wild-type and arrested at the globular stage (Figure 2B and Figure S1); mature grains were unfilled (Figure 2A). Compared with the empty vector controls, the average length of silique was reduced by approximately 33% for *nrpc2-1* and 26% for *nrpc2-2* (Figure 2C). The seed number per silique was reduced by approximately 39% and 35% for *nrpc2-1* and *nrpc2-2*, respectively (Figure 2D).

In *nrpc3-1* and *nrpc3-2,* the relative expression level of *NRPC3* was reduced by 73% and 86% compared with control lines, respectively (Figure 2E). The development of mutant embryos arrested at the early cotyledon stage (Figure 2B and Figure S1) and mature grains consisted only of a seed coat (Figure 2A). Compared with those of the empty vector controls, the silique average length of *nrpc3-1* and *nrpc3-2* was reduced by approximately 10% and 31% (Figure 2C), and the seed number per silique was reduced by approximately 20% and 42%, respectively (Figure 2D).

The relative expression level of *NRPC8* was notably reduced in *nrpc8-1* and *nrpc8-2* (Figure 2E). The mutant embryos arrested at the preglobular stage (Figure 2B and Figure S1) and mature grain had only the seed coat (Figure 2A). The silique average length of *nrpc8-1* and *nrpc8-2* was reduced by approximately 34% and 15% compared to wild-type (Figure 2C), and the seed number per silique was reduced by approximately 38% and 30%, respectively (Figure 2D).

In *nrpabc1-1* and *nrpabc1-2,* the relative expression level of *NRPABC1* was down-regulated by 88% and 39%, respectively, compared with the wild-type (Figure 2E). The development of mutant embryos was slower than that of the wild-type, and they arrested at the early cotyledon stage (Figure 2B and Figure S1). Mature grains were smaller than in the wild-type (Figure 2A). The average silique length of *nrpabc1-1* and *nrpabc1-2* was reduced by approximately 21% and 17%, respectively, compared with the empty vector controls (Figure 2C); meanwhile, the seed number per silique was reduced by approximately 31% and 30%, respectively (Figure 2D).

In *nrpabc2-1,* the relative expression levels of *NRPABC2-1* and *NRPABC2-2* were reduced by 74% and 99%, respectively, and in *nrpabc2-2,* the relative expression levels of *NRPABC2-1* and *NRPABC2-2* were reduced by 32% and 82%, respectively (Figure 2E). The mutant embryos arrested at the early cotyledon stage (Figure 2B and Figure S1), and the mature grains were smaller than that of the wild-type (Figure 2A). In *nrpabc2-1* and *nrpabc2-2*, the average length of silique was reduced by approximately 17% and 27%, respectively, when compared with the empty vector controls (Figure 2C), while the seed number per silique was reduced by approximately 29% and 37%, respectively (Figure 2D).

Knockdown of *NRPC2* and *NRPC8* led to the arrest of embryo development at the globular stage, while knockdown of *NRPC3*, *NRPABC1*, and *NRPABC2* arrested the embryo at the early cotyledon stage. Meanwhile, the silique length and seed number were reduced in all of these RNAPIII subunit knockdown lines. These results demonstrate that *NRPC2*, *NRPC3*, *NRPC8*, *NRPABC1*, and *NRPABC2* control seed development in Arabidopsis.

### 2.4. NRPC2, NRPC3, NRPC8, and NRPABC2 Interact with Other Subunits of the RNAPIII Complex

**Figure 3:**
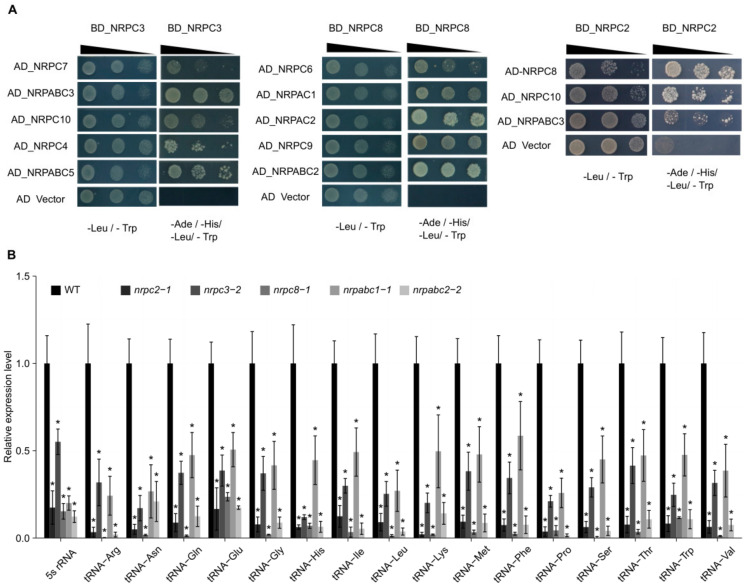
Interactions between RNAPIII subunits and expression of 16 tRNAs and 5S rRNA in wild-type (WT) and different subunit knockdown lines seeds at 10 DAF. (**A**) Results of yeast two-hybrid analysis of the interactions between Arabidopsis RNAPIII subunits. Yeast cultures at three different dilutions (10, 10 21, and 10 22) were grown on synthetic defined (SD) medium lacking Trp, Leu, His, and Ade (right) and lacking Trp and Leu (left, as control), respectively. AD, activating domain; BD, binding domain. (**B**) qRT-PCR analysis of 17 tRNA and 5S rRNA genes in 10 DAF seeds of wild-type and different subunit knockdown lines. All expression levels are normalized to that of *ACTIN*. Three replicates for each sample were performed, and data are means ± SD. Asterisks indicate a significant difference in the Student’s *t*-test (“*” *p* < 0.05).

With 17 subunits, RNAPIII is the largest of the three RNA polymerases. It comprises the conserved 10-subunit core, a 2-subunit peripheral stalk (NRPC8-NRPC9), an NRPC4-NRPC5 heterodimer, and the NRPC3–NRPC6–NRPC7 heterotrimer [4,25]. To further investigate the nature of these subcomplexes, we applied a yeast-two-hybrid (Y2H) analysis to screen for the relationships between different subunits. We found that NRPC2 physically interacts with other subunits of the RNAPIII complex, including NRPC8, NRPC10, and NRPABC3. Meanwhile, NRPC7, NRPC10, NRPC4, NRPABC3, and NRPABC5 were confirmed to participate in a protein–protein interaction with NRPC3, and NRPC8 physically interacted with NRPC6, NRPC9, NRPAC1, NRPAC2, and NRPABC2 (Figure 3A). Together these results show that NRPC2, NRPC3, NRPC8, and NRPABC2 all interact with other subunits of the RNAPIII complex.

### 2.5. Expression of tRNAs and 5S rRNA Is Disrupted in nrpc2, nrpc3, nrpc8, nrpabc1, and nrpabc2 Mutant Seeds

To elucidate the effects of NRPC2, NRPC3, NRPC8, NRPABC1, and NRPABC2 on RNAPIII transcription, we analyzed the biogenesis of tRNAs and 5S rRNA in *nrpc2-1*, *nrpc3-2*, *nrpc8-1*, *nrpabc1-1* and *nrpabc2-2* seeds, respectively. The transcript levels of 5S rRNA and 16 tRNAs decreased fivefold in *nrpc2-1* seeds at 10DAF. In particular, the expression of 14 tRNAs (except tRNA-Glu and tRNA-Ile) reduced by more than tenfold, relative to wild-type seeds (Figure 3B). In *nrpc3-2* 10 DAF seeds, the transcript levels of 5S rRNA were down-regulated by approximately twofold, while those of the 16 tRNAs decreased more than twofold. In *nrpc8-1* 10 DAF seeds, the relative expression level of 5S rRNA was reduced by 85%, and the expression of 15 tRNAs were down-regulated by more than tenfold, relative to wild-type seeds. The transcript level of 5S rRNA was reduced by 80% in *nrpabc1-1* 10 DAF seeds, and 15 of the 16 tested tRNAs were down-regulated by more than twofold relative to wild-type seeds. In 10 DAF *nrpc3-2* seeds, the transcript levels of 5S rRNA and 16 tRNAs were reduced by more than fivefold relative to wild-type seeds. These results indicate that NRPC2, NRPC3, NRPC8, NRPABC1, and NRPABC2 regulate the transcription of 5S rRNA and tRNAs in Arabidopsis seeds (Figure 3B).

### 2.6. nrpc2, nrpc3, nrpc8, nrpabc1, and nrpabc2 Mutant Seeds Show Transcriptomic Alterations

**Figure 4:**
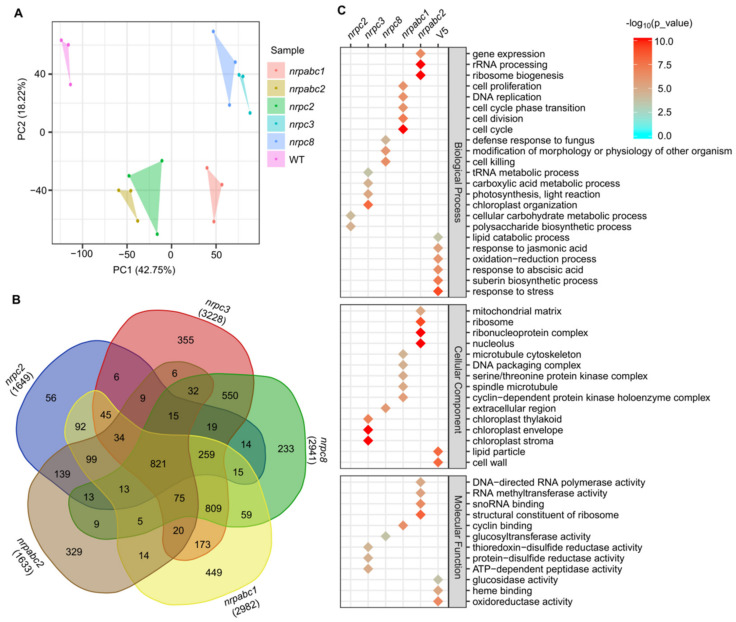
Global transcriptome analysis based on RNA-seq data in 10 DAF seeds of wild-type and different subunit knockdown lines. (**A**) PCA comparing the expressed genes of different samples. (**B**) Numbers of DEGs in the seeds of different subunit knockdown lines compared with the wild-type. (**C**) GO classification for genes with differential expression in the seeds of different subunit knockdown lines compared with the wild-type.

To explore the molecular basis of how these different RNAPIII subunits regulate seed development in Arabidopsis as described above, we performed RNA-seq to generate transcriptome profiles. We sampled Arabidopsis seeds at 10 DAF from *nrpc2-1*, *nrpc3-2*, *nrpc8-1*, *nrpabc1-1*, *nrpabc2-2,* and wild-type lines (three biological replicates from each genotype). Principal component analysis (PCA) revealed that the six samples could be clearly assigned to four groups (wild-type; *nrpabc1*; *nrpc2*; *nrpabc2*; *nrpc3*; and *nrpc8*), and transcriptome characteristics were highly correlated between different biological replicates of each genotype sample (Figure 4A). We identified 1649 DEGs (*p*-value < 0.01 and abs(log_2_(Fold-Change) > 1) between *nrpc2* and the wild-type; the number of DEGs between *nrpc3* and the wild-type was 3228, almost twice as much as *nrpc2* between *nrpc2* and the wild-type. Compared to the wild-type, 2941, 2982, and 1633 genes were significantly differentially expressed in *nrpc8*, *nrpabc1*, and *nrpabc2*. We found 821 common DEGs in all five genotypes of mutant seeds and 56, 355, 233, 449, and 329 unique DEGs in *nrpc2*, *nrpc3*, *nrpc8*, *nrpabc1,* and *nrpabc2* seeds, respectively (Figure 4B).

Based on the identified DEGs, we performed GO classification to examine the molecular changes involved in these different mutants. GO terms that were preferentially enriched in common DEGs were associated with pathways such as a response to stress, response to jasmonic acid and abscisic acid, oxidoreductase activity, heme binding and lipid particle. Dominant enriched GO terms in *nrpc2* unique DEGs were the cellular carbohydrate metabolic process and polysaccharide biosynthetic process. Significantly enriched GO terms in DEGs unique to *nrpc3* were: the tRNA metabolic process; carboxylic acid metabolic process; ATP-dependent peptidase activity; and chloroplast stroma. GO terms associated with cell death: defense response to fungus; modification of morphology or physiology of the other organism. Glucosyltransferase activity was enriched in DEGs unique *nrpc8*. GO terms related to the cell cycle: cell division; cell proliferation; DNA replication; cyclin binding; cyclin-dependent protein kinase holoenzyme complex. DNA packaging complexes were enriched in DEGs unique to *nrpabc1*. GO terms associated with rRNA processing: ribosome biogenesis; gene expression; ribonucleoprotein complex; structural constituent of ribosome. DNA-directed RNA polymerase activity was enriched in DEGs unique to *nrpabc2* (Figure 4C). These results suggest that storage reserve filling and hormone balance were destroyed in these subunit knockdown seeds; however, knocking down different subunits appeared to influence distinct pathways.

**Figure 5:**
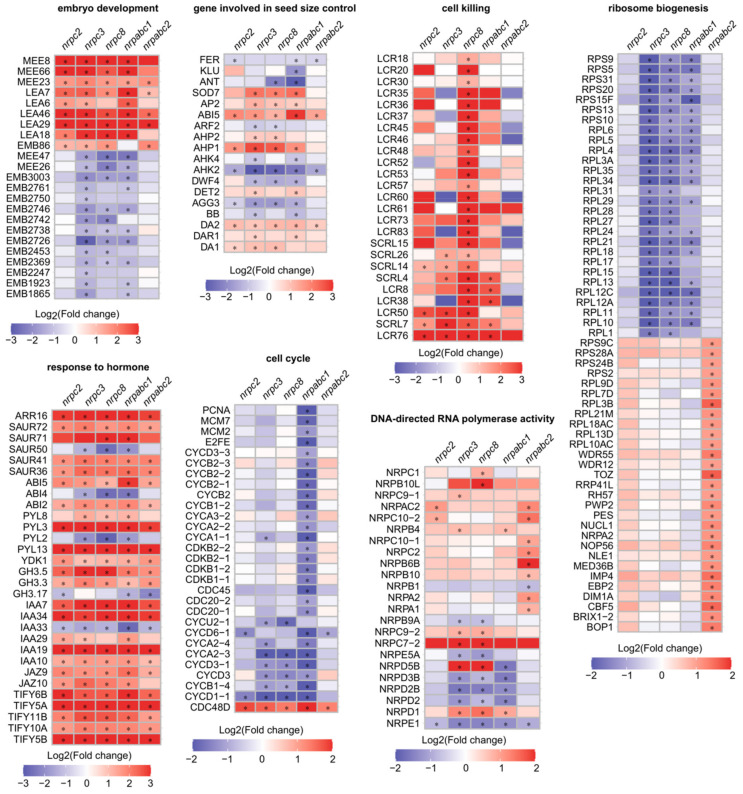
Heat maps of DEGs functioning in selected disrupted pathways (“*” *p* < 0.01, absolute value of log_2_(fold change) > 1).

Further analysis of these DEGs revealed notable effects on genes with functions in the seed development regulation. Specifically, there were 23 seed development-related proteins with altered expressions in at least one mutant (Figure 5). Eighteen seed size regulators, which have been confirmed to influence seed growth and belong to different signaling pathways, were differentially expressed in at least one mutant (Figure 5). Thirty genes associated with the biosynthesis and signaling of phytohormones such as ABA, indole-3-acetic acid (IAA), auxin and JA were identified as DEGs in all mutant lines, and most of these genes were up-regulated in mutant seeds (Figure 5). Genes encoding cell-cycle-dependent proteins also featured prominently among the identified DEGs, including five A-type cyclins, five B-type cyclins, four D-type cyclins, and four cyclin-dependent kinases. Most of these cell-cycle-dependent proteins were down-regulated in *nrpabc1* seeds. Twenty-five genes encoding putative defensin-like proteins were up-regulated in *nrpc8* seeds, including 20 low-molecular-weight cysteine-rich proteins and 5 S-locus cysteine-rich proteins. Fifty-seven ribosome biogenesis factors featured among the identified DEGs; 29 of them were significantly up-regulated in *nrpabc2* seeds, and the others were significantly down-regulated in *nrpc3, nrpc8*, and *nrpabc1* seeds. We identified 23 instances of DNA-directed RNA polymerase activity associated with DEGs, most with different expression patterns in different mutant seeds (Figure 5). These results demonstrate that several regulatory pathways of seed size control, such as phytohormones, ubiquitin-proteasome pathway, and G-protein signaling factor and transcriptional regulatory pathways, were altered in RNAPIII subunit knockdown lines. Meanwhile, the insufficient accumulation of the total RNAPIII at different stages of seed development resulted in the identification of several unique DEGs belonging to distinct pathways in seeds of different subunits mutants.

### 2.7. Co-Expression Network Analysis Identified Genes Related to Seed Development

**Figure 6:**
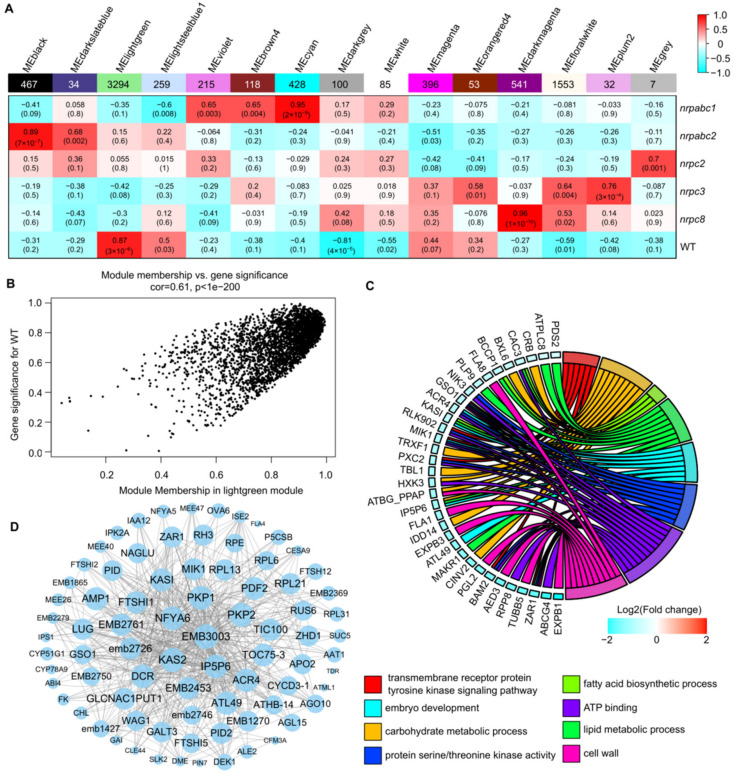
WGCNA of the transcripts in wild-type and different subunit knockdown lines. (**A**) Association between modules and traits. The legend indicates the correlation. Each cell shows the number of corresponding correlations and the *p*-values. (**B**) Module membership versus gene significance in the MElightgreen module. (**C**) GO classification of hub genes in the MElightgreen module. (**D**) Network analysis of the proteins related to seed development or embryo development in the MElightgreen module.

To identify candidate hub genes correlating to the developmental arrest of Arabidopsis seeds from these RNAPIII subunit knockdown lines, we used weighted gene co-expression network analysis (WGCNA). This is a powerful tool for identifying which sets of genes are associated with phenotypes using RNA-seq data. A total of 7582 DEGs were retained for the constructed co-expression network, and 15 distinct modules were identified based on the pairwise correlations of gene expression levels across all samples when the soft thresholding power was set as 14 (Figure 6A and Figure S2). These modules were labeled in different colors, and the MElightgreen module shows the significant and positive correlations with the seed phenotype (*r* = 0.87, *p*-value = 3 × 10^−6^) (Figure 6A and Figure S3). The MElightgreen module is the largest module, containing 43.45% of all DEG genes used in this analysis. The gene significance (GS) and module membership (MM) had a high correlation and very small *p*-values for all genes in this module (cor = 0.61, *p* < 1 × 10^−200^) (Figure 6B).

Based on the cut-off criteria (abs(MM) + abs(GS) > 1.86), 100 genes with high connectivity in the MElightgreen module were identified as hub genes. GO enrichment analysis of these hub genes revealed enriched GO related to the transmembrane receptor protein tyrosine kinase signaling pathway, embryo development, carbohydrate metabolic process, protein serine/threonine kinase activity, fatty acid biosynthetic process, ATP binding, lipid metabolic process, and cell wall (Figure 6C). Eighty-two of the hub genes were annotated as seed development or embryo development-related proteins, including 11 embryo-defective proteins, 3 maternal effect embryo arrest proteins, 4 ribosomal proteins, etc. (Figure 6D). We found that the degree of these proteins varied from 997 to 3130 in the MElightgreen module network; EMB3003, encoded by a 2-oxoacid dehydrogenase acyltransferase family protein, has the largest degree in this network. KAS2, also known as fatty acid biosynthesis1 (fab1), has a degree of 3082 in this network. The *fab1* mutant has increased levels of the saturated fatty acid 16:0, resulting in embryo arrest at the early embryo development stage [26]. Plastidic pyruvate kinase (PKPs) catalyze the synthesis of pyruvate and ATP; PKP1 and PKP2 have degrees of 3042 and 3038 in this network, respectively. In the *pkp2* mutant, embryo elongation appeared to be drastically retarded compared to the wild-type, and this developmental defect was even more pronounced in the *pkp1pkp2* double mutant [27] (Figure 6D). This suggests that the hub genes identified here likely play critical roles in Arabidopsis seed development.

## 3. Discussion

Previous reports have shown that *ZmNRPC2* controls seed development by regulating cell proliferation and endoreduplication in maize endosperm [22]. GL6 interacts with RNAPIII subunit C53 (NRPC4) and transcription factor class C1 (TFC1), regulating the expression of genes involved in rice grain development to promote cell proliferation in young panicles and grains [28]. Another PLATZ protein, FL3, has a similar function to GL6, interacting with the RNAPIII subunit 53 (NRPC4) and transcription factor class C1 (TFC1) to regulate endosperm development and storage reserve filling in maize [29]. In this study, we demonstrated that the knockdown of *NRPC2*, *NRPC3*, *NRPC8*, *NRPABC1*, or *NRPABC2* caused RNAPIII defects, leading to reduced expression of RNAPIII-transcribed 5S rRNA and 16 tRNAs. The knockdown of the genes encoding these subunits in RNAPIII had a pronounced effect on seed development in Arabidopsis; specifically, embryo development was slower than in the wild-type, and embryos arrested at the globular or cotyledon stages in these mutant lines. Meanwhile, the average silique length and the seed number per silique was reduced (Figure 2). These findings indicate that RNAPIII controls seed development in Arabidopsis.

Previous reports have shown that seed development is controlled by several signaling pathways, including the ubiquitin–proteasome, G-protein signaling, phytohormones, transcriptional regulatory factors pathways [1,2]. The ubiquitin receptor, *DA1,* functions synergistically with E3 ubiquitin ligases, *DA2* and *BIG BROTHER (BB)/ENHANCER OF DA1 (EOD1),* to regulate seed size by restricting cell proliferation in developing seeds [30,31,32]. *DA1* was up-regulated in *nrpc2*, *nrpc3*, and *nrpc8* seeds, and *DA2* was up-regulated in *nrpc2*, *nrpc3*, *nrpc8*, *nrpabc1*, and *nrpabc2* seeds. Meanwhile, *BB* was down-regulated in *nrpc3* and *nrpabc1* seeds. G-protein signaling transmits a signal to downstream effectors via the heterotrimeric G-protein complex, playing an integral role in mediating multiple signaling pathways in plants. Mutations in atypical *Gγ (AGG3)*, encoding a subunit of the G-protein complex, causes small seeds and organs by decreasing cell proliferation [33,34], while overexpression of *AGG3* promotes seed and organ growth [35]. *AGG3* was down-regulated in *nrpc2*, *nrpc3*, *nrpc8*, and *nrpabc1* seeds. Several phytohormones have been suggested to have important roles in seed growth. For example, brassinosteroids (BR) positively regulate seed development [36]; auxin controls seed size by regulating cell proliferation [37]; cytokinins influence seed growth and development [38]. Over-expression of *DWARF 4 (DWF4)* results in increased BR levels, leading to an increase in seed yield [39]. Loss of the *AUXIN RESPONSE FACTOR2 (ARF2)* function activates cell division and leads to enlarged seed coats [37]. *AINTEGUMENTA (ANT)* acts downstream of ARF2, and over-expression of ANT enlarges seed size by increasing the cell number [40]. *ARABIDOPSIS HISTIDINE KINASES (AHKs)* act as cytokinin receptors to control steady-state cytokinin levels, regulating embryo size. *ARABIDOPSIS HISTIDINE PHOSPHOTRANSFER PROTEINS (AHPs)* encode positive regulators of cytokinin signaling, which affects seed development in *Arabidopsis* [38,41]. It is noteworthy that the expression levels of phytohormone biosynthesis, or signaling-related genes *DWF4*, *DET2*, *ARF2*, *ANT*, *AHP1*, *AHP2*, *AHK4*, *AHK2,* and others, were changed in one or more RNAPIII subunit mutants (Figure 5). Meanwhile, 30 phytohormone biosynthesis and signaling-related genes, 5 MEEs, 5 LEAs, and 13 EMBs related genes, were identified as DEGs in these mutants (Figure 5). Therefore, RNAPIII is involved in the transcriptional regulation of seed development signaling pathways; knockdown of these genes, encoding the NRPC2, NRPC3, NRPC8, NRPABC1, and NRPABC2 subunits, disrupts these signaling pathways, thereby affecting seed development.

It is remarkable that seven hub genes in our MElightgreen module co-expression network are related to embryo development (*GSO1*, *ZAR1, KASI*, *ATL49*, *ACR4*, *CVP2*, and *MIK1*) (Figure 6C); *GSO1* encodes a receptor-like kinase (RLK), which acts as both a signal receptor and signal transducer in ligand mediated communication between cells to regulate seed development in Arabidopsis [42]; *ZAR1* encodes a member of the RLK/Pelle kinase family, and the asymmetric division of zygotes is impaired in *zar1* mutants [43]; *KASI* encodes β-ketoacyl-[acyl carrier protein] synthase and catalyzes the elongation of fatty acid (FA) synthesis, and embryo development is disrupted before the globular stage in *kasi* mutants [44]. Meanwhile, 82 seed development or embryo development-related genes are enriched in our MElightgreen module. Previous studies have shown that some of these genes regulate seed development in Arabidopsis, including *Fatty Acid Biosynthesis1* (*KAS2/FAB1*) [26], plastidic pyruvate kinase *PKP1* and *PKP2* [27], *NF-YA family member6* (*NFYA6*) [45], carboxypeptidase *ALTERED MERISTEM PROGRAM 1* (*AMP1*) [46], *β-Ketoacyl-[acyl carrier protein] synthase I* (*KASI*) [44], and the homeobox genes *MERISTEM LAYER1* (*ATML1*) and *PROTODERMAL FACTOR2* (*PDF2*) [47]. These genes have many degrees in the MElightgreen module co-expression network (Figure 6D). The hub genes identified from the gene co-expression network of RNAPIII subunit knockdown seeds provides a more detailed understanding of the seed development regulatory network.

Different subunits of RNAPIII control different stages of RNAPIII transcription. *NRPC6 tWH* mutants can influence protein and nucleic acid interactions and undermine RNAPIII transcription initiation and elongation [11]; NRPC3, NRPC2, and NRPC1 are important for the assembly of the RNAPIII holoenzyme [48,49]; and NRPC7 and NRPC3 are important in the initiation of RNAPIII transcription [9,10]. Differentiation in the functions of different RNAPIII subunits have been reported by previous studies. A partial loss of function of RNAPIII subunit NRPC7 leads to pleiotropic defects such as shorter siliques and roots, delay in flowering time, bumpy sepal texture, and irregular sepal shape [50]. NRPC2 regulates cell proliferation and endoreduplication to control endosperm development in maize [22]. The PLANTZ protein FL3 participates in the regulation of RNAPIII transcription machinery and regulates storage reserve filling in maize [29]. Intriguingly, the expression pattern of several RNAPIII subunits were distinct from each other in Arabidopsis (Figure 1), and seeds arrested at different development stages. in different RNAPIII subunit knockdown lines (Figure 2). Meanwhile, we identified numerous unique DEGs in different RNAPIII subunit knockdown seeds, and these unique DEGs belong to different pathways. Specifically, DEGs unique to *nrpabc1* were significantly enriched in ribosome biogenesis; cell cycle-related genes were significantly enriched in DEGs unique to *nrpabc2*; DEGs unique to *nrpc3* were enriched in chloroplast organization; cell death and defense response to fungus-related genes were enriched in DEGs unique to *nrpc8*; and DEGs unique to *nrpc2* were enriched in carbohydrate metabolic processes (Figure 4C and Figure 5). These findings indicate that differential expression levels of different sub-complexes in subunits of RNAPIII across seed developmental stages result in insufficient accumulation of total RNAPIII, which corresponds to the different phenotypes observed in different subunit knockdown seeds.

In conclusion, the results of this study indicate that knocking down expression of RNAPIII subunits NRPC2, NRPC3, NRPC8, NRPABC1, and NRPABC2 represses RNAPIII transcription machinery activity and down-regulates the expression of 5S rRNA and tRNAs. This resulted in seed developmental arrest at an early stage in Arabidopsis. By analyzing the transcriptome of these mutants, we identified several DEGs, some of which have been confirmed to control Arabidopsis seed development in previous studies, while some act as hub genes in the seed development co-expression network. Moreover, we found that mutant seeds arrest at different stages in different RNAPIII subunit knockdown lines, and DEGs unique to different subunit mutant seeds belong to distinct pathways, showing function differentiation of different subunits in RNAPIII. Although the detailed mechanism by which RNAPIII regulates the expression of these seed development regulators remains unclear, our data contributes toward the understanding of the RNAPIII transcription machinery regulating seed development in plants.

## 4. Materials and Methods

### 4.1. Plant Material

In the *Arabidopsis thaliana* Columbia-0 background, coding sequences (CDS) of gene encoding RNAPIII subunits were amplified and inserted into the pCAMBIA1300 vector under the control of the cauliflower mosaic virus (CaMV) 35S promoter using SmaI restriction sites. The recombinant plasmid was then transformed into *Agrobacterium tumefaciens* strain GV3101 using the floral dip method. T_0_ seeds were sterilized in 75% ethanol for 2 min. Transgene selection was performed on solid Murashige and Skoog medium supplemented with 50 µg/mL kanamycin. Resistant seedlings were transferred to soil and grown at a constant temperature of 22 °C under long-day conditions (16-h light/8-h dark). Independent T_2_ transgenic lines were obtained using PCR selection. The primers used for the plasmid construct are listed in Supplemental Table S1.

### 4.2. Cytological Observations

Seeds at 15 days after flowering (DAF) were collected and fixed overnight in 4% paraformaldehyde (Sigma, Santa Clara, CA, USA), then dehydrated in an ethanol gradient series (30, 50, 70, 85, 95, and 100% ethanol), and embedded in Paraplast Plus (Sigma, Santa Clara, CA, USA). The sample blocks were sectioned into 8-μm slices using a Leica RM2265 microtome (Leica Microsystems, Wetzlar, Hesse-Darmstadt, Germany) and stained with 0.5% toluidine blue. Images were captured using a Leica MZFLIII microscope (Leica Microsystems, Wetzlar, Hesse-Darmstadt, Germany).

### 4.3. Yeast Two-Hybrid Assay

For the yeast two-hybrid (Y2H) assays, the CDS of the RNAPIII subunits were cloned into the pGBKT7 plasmid and transformed into the yeast strain Y2HGold. Meanwhile, these CDS were also inserted into the pGADT7 plasmid and transformed into the yeast strain Y187. All yeast experiments were performed as referenced in Clontech’s Yeast Protocols Handbook. The primers used for Y2H are listed in Supplemental Table S1.

### 4.4. RNA Extraction and qRT-PCR

Seeds from the wild-type and RNAPIII subunit knockdown lines were harvested 10 days after flowering (DAF), quickly frozen in liquid nitrogen, and ground to a fine powder with a mortar and pestle. Total RNA was extracted with 1 mL TRIzol reagent (Thermo Fisher Scientific, Waltham, MA, USA), according to the manufacturer’s instructions. After isopropanol precipitation, the RNA was resuspended in 50 µL RNase-free water and treated with RNase-free DNase I. For reverse transcription, 1 µg total RNA was used in each 20-µL reaction, and the first cDNA strand was synthesized using SuperScript II (Invitrogen) and hexamer primers according to the manufacturer’s instructions. SYBR Green (Bio-Rad, Hercules, CA, USA) was added to the PCR reaction according to the guidelines. Quantitative RT-PCR analysis was carried out on three independent RNA samples and *ZmActin1* as an internal control using a Bio-Rad CFX96 Touch Real-time PCR detection system. The relative expression of mRNA was calculated using the 2^−∆∆Ct^ method [51]. The expression of 5S rRNA and tRNAs was analyzed by qRT-PCR. The primers used for qRT-PCR are listed in Supplemental Table S1.

### 4.5. RNA-Seq Analysis

Total RNA was extracted from wild-type and mutant seeds using TRIzol reagent and then subjected to cleanup and DNase I treatment using a Qiagen RNeasy Mini Kit, according to the manufacturer’s protocol. Three independent biological replicates from three different plants were performed. cDNA libraries were constructed following the standard Illumina protocol and sequenced on the Illumina NovaSeq platform by Novogene. The sequence reads were trimmed using Trimmomatic (version 0.33) and mapped to the TAIR10 reference genome using HISAT2 (version 2.1.0). StringTie (version 1.3.3b) was employed to reconstruct the transcripts and estimate gene expression levels [52]. HTSeq (version 0.6.1) was used to count the number of reads per gene. DEGs were identified using DESeq2, with significant DEGs being those with a *p*-value of differential expression above the threshold (*p* < 0.01, absolute value of log_2_(fold change) > 1). Conjoint Gene Ontology (GO) and Kyoto Encyclopedia of Genes and Genomes (KEGG) enrichment results were generated using the website software “AgriGO” (http://systemsbiology.cau.edu.cn/agriGOv2/ assessed on 14 October 2021) [53]. Analysis of RNA-seq data quality is provided in Supplemental Tables S2 and S3.

### 4.6. Weighted Gene Co-Expression Network Analysis (WGCNA)

A gene co-expression network was constructed using the R package WGCNA [54]. DEGs in seeds from mutant lines were used for analysis, with an adjacency matrix constructed based on normalized fragments per kilobase of transcript, per million mapped read (FPKM) values. Modules were obtained using the automatic network construction function blockwiseModules with the parameters: softpower was 12, TOM-Type was adjacency, minModuleSize was 30, and mergeCutHeight was 0.225. Co-expression networks were visualized using Cytoscape software [55].

## Figures and Tables

**Table 1:** The list of Arabidopsis RNAPIII subunits and their homologs.

*S. cerevisiae*	Accession Number	*H. sapiens*	Accession Number	*Arabidopsis thaliana*	Gene ID
RNA Pol III Subunits		RNA Pol III Subunits		RNA Pol III Subunits	
C160 (β-like)	P04051	HsNRPC1	AAB86536	AtNRPC1	AT5G60040
C128 (β-like)	AAB59324	HsNRPC2	AY092084	AtNRPC2	AT5G45140
C82	CAA45072	HsNRPC3	NP_006459/	AtNRPC3	AT3G49000
			XP_034604		
C53	P25441	HsNRPC4	AY092086	AtNRPC4-1	AT5G09380
				AtNRPC4-2	AT4G25180
C37	NP_012950	HsNRPC5	AY092085	AtNRPC5	AT5G49530
C34	P32910	HsNRPC6	NP_006457/	AtNRPC6	AT5G23710
			XP_009639		
C31	P17890	HsNRPC7	AAB63676/	AtNRPC7-1	AT4G01590
			XP_036456	AtNRPC7-2	AT4G35685
C25	P35718	HsNRPC8	AY092087	AtNRPC8	AT1G06790
C17	P47076	HsNRPC9	AAC25992	AtNRPC9-1	AT5G62950
				AtNRPC9-2	AT3G28956
C11	AAD12060	HsNRPC10	NP_057394	AtNRPC10-1	AT1G01210
				AtNRPC10-2	AT4G07950
AC40 (α-like)	P07703	HsNRPAC1	NP_004866	AtNRPAC1	AT1G60620
AC19 (α-like)	P28000	HsNRPAC2	NP_057056	AtNRPAC2	AT2G29540
ABC27	P20434	HsNRPABC1	P19388	AtNRPABC1	AT3G22320
ABC23 (ω-like)	AAA34989	HsNRPABC2	P41584	AtNRPABC2-1	AT5G51940
				AtNRPABC2-2	AT2G04630
ABC14.5	CAA37383	HsNRPABC3	P52434	AtNRPABC3-1	AT1G54250
				AtNRPABC3-2	AT3G59600
ABC10α	AAA64417	HsNRPABC4	P53803	AtNRPABC4	AT5G41010
ABC10β	P22139	HsNRPABC5	P52436	AtNRPABC5	AT1G11475

## Data Availability

The data that support the findings of this study are openly available in the NCBI Sequence Read Archive at identification SRA accession number PRJNA753225.

## Supplementary Materials

The following are available online at https://www.mdpi.com/article/10.3390/ijms222111314/s1.
