# Supplementary material for: Knockdown NRPC2, 3, 8, NRPABC1 and NRPABC2 Affects RNAPIII Activity and Disrupts Seed Development in Arabidopsis
Source: Int J Mol Sci. 2021 Oct 20;22(21):11314. doi: 10.3390/ijms222111314 (PMC8583208; doi:10.3390/ijms222111314)
Supplement: Supplementary file 1 [file ijms-22-11314-s001.zip › ijms-1402296-supplementary.pdf]

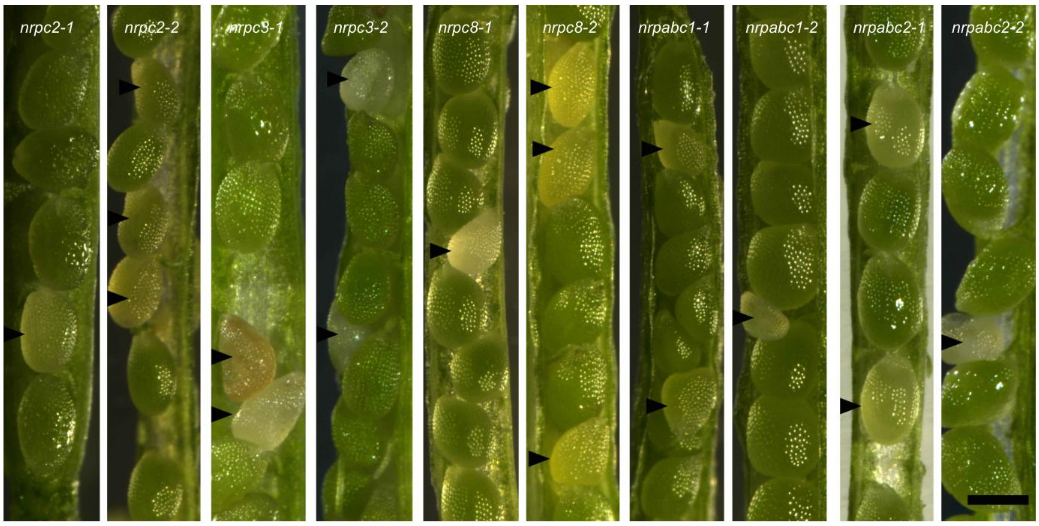

Supplemental Figure S1. Phenotypic features of different subunits knockdown seeds at 15DAF (black arrow indicates mutant seeds, Scale bar = 300 um).

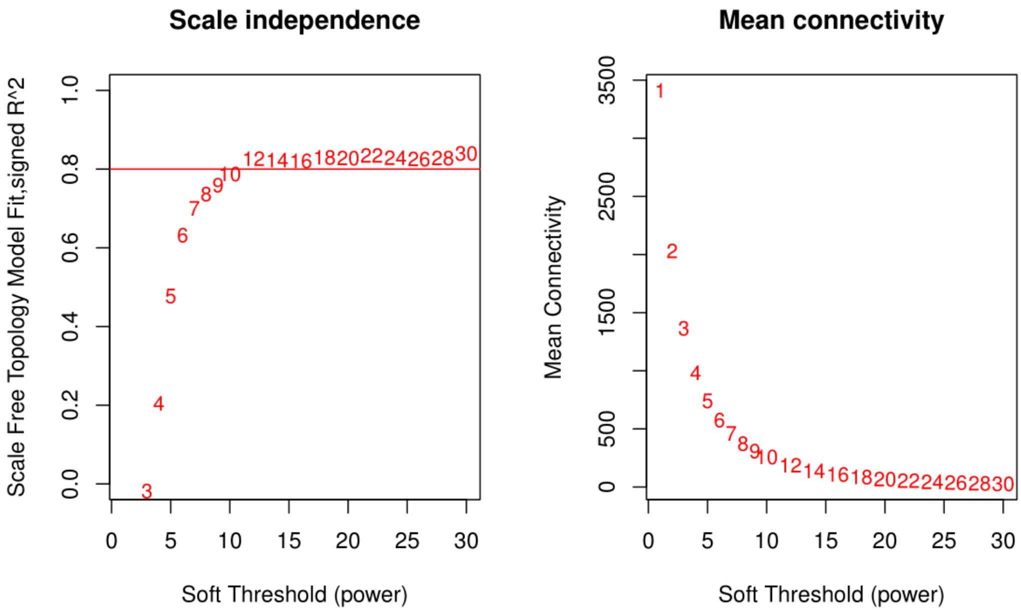

Supplemental Figure S2. Soft-thresholding powers test in WGCNA.

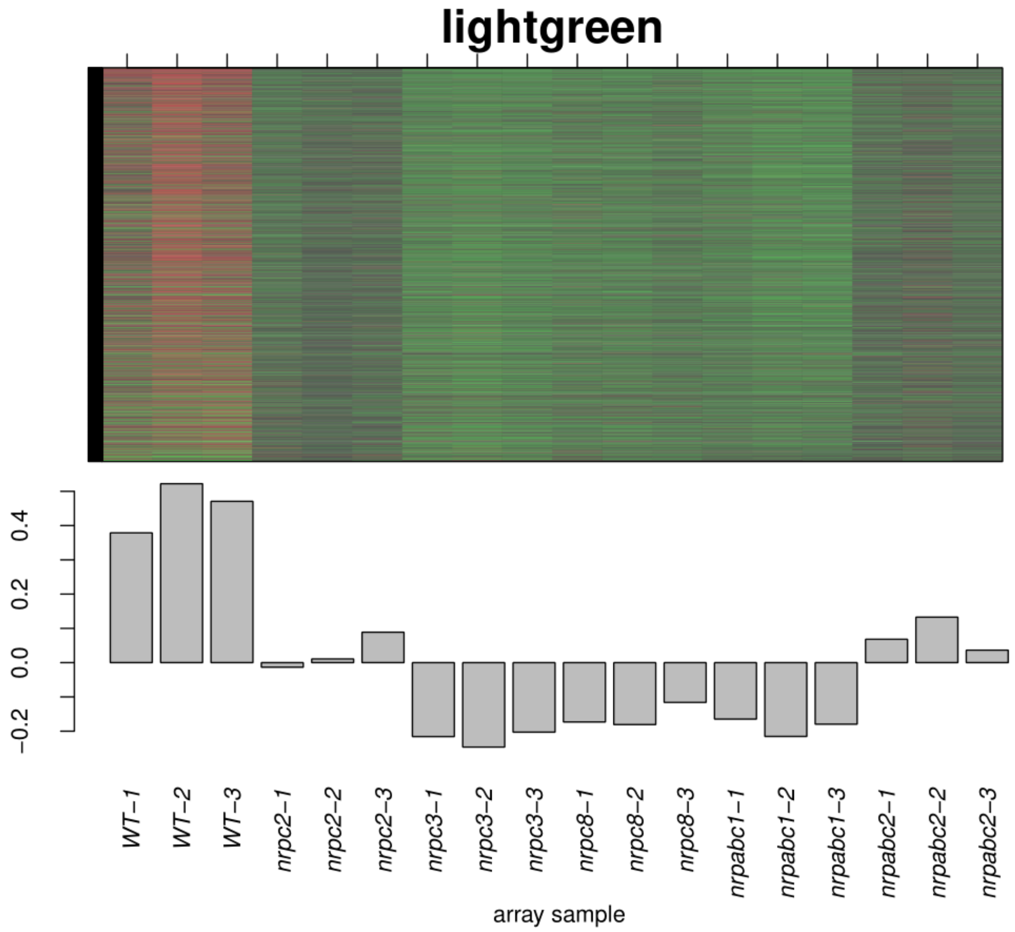

Supplemental Figure S3. The co-expressed genes are shown in heatmap and bar graph in the lightgreen module.

Supplemental Table S1. The primers used in this study.

qNRPC2-F	AGCGATGGGACTTGATCAAG	qRT_PCR
qNRPC2-R	TGCTTCACAAGTCCTCTCAC	qRT_PCR
qNRPC3-F	TCTCCATTGAACCTCCTG	qRT_PCR
qNRPC3-R	CTTGGAGTCAAGTTCTTCG	qRT_PCR
qNRPC8-F	CTCTCGAAGATGCCATTAAG	qRT_PCR
qNRPC8-R	TATAGGTCGCAGCACCATC	qRT_PCR
qNRPABC1-F	AGGCGGAAATGCTAGTGAAC	qRT_PCR
qNRPABC1-R	GTCACTTGGATCCTTGGAAG	qRT_PCR
qNRPABC2-1-F	GATTACAACGACGTCGATG	qRT_PCR
qNRPABC2-1-R	CAATAGGCTCACCATTGAC	qRT_PCR
qNRPABC2-2-F	TCGACTTTAATGGCTGACG	qRT_PCR
qNRPABC2-2-R	CTGTTTCCAAAGGGTCAAC	qRT_PCR
qAT-Arg-F	CCTGTAGCTCAGTGGATAG	qRT_PCR
qAT-Arg-R	AGGTAGGGGTCGAACCTAC	qRT_PCR
qAT-Ala-F	GGATGTAGCTCAGATGGTAG	qRT_PCR
qAT-Ala-R	GAGATGCGGGGTATCGATC	qRT_PCR
qAT-Asn-F	GCTGGAATAGCTCAGTTGG	qRT_PCR

qAT-Asn-R	CTAGAAGGAGGGGTCGAAC	qRT_PCR
qAT-His-F	GGTGGCTGTAGTTTAGTGG	qRT_PCR
qAT-His-R	TGTGGCTGCTGGGATTCG	qRT_PCR
qAT-Glu-F	CCGTTGTAGTCTAGCTGGTC	qRT_PCR
qAT-Glu-R	TCCGTTGCCGGGACTCGAAC	qRT_PCR
qAT-Ile-F	GGCCTATTAGCTCAGTTGG	qRT_PCR
qAT-Ile-R	TGGTGGCCTATACAGGGATC	qRT_PCR
qAT-Lys-F	CCGTCTTAGCTCAGTGGTAG	qRT_PCR
qAT-Lys-R	TCTGTGGGGATCGAACCCAC	qRT_PCR
qAT-Met-F	TCAGAGTGGCGCAGCGGAAG	qRT_PCR
qAT-Met-R	GGTATCAGAGCCAGGTTTCG	qRT_PCR
qAT-Phe-F	ATAGCTCAGTTGGGAGAGCG	qRT_PCR
qAT-Phe-R	GGTGAGCGTGGATCGAACAC	qRT_PCR
qAT-Pro-F	GCATTTGGTCTAGTGGTATG	qRT_PCR
qAT-Pro-R	CATTCCGAGAATCGAACTC	qRT_PCR
qAT-Ser-F	ACGTGCCGGAGTGGTTATC	qRT_PCR
qAT-Ser-R	TGAACGGCAGGATTCGAAC	qRT_PCR
qAT-Thr-F	CGTATAGCTCAGTGGTAGAG	qRT_PCR
qAT-Thr-R	ACACGCAGGATCGAACTAC	qRT_PCR
qAT-Trp-F	GATCCGTGGCGCAATGGTAG	qRT_PCR
qAT-Trp-R	TGGTGAACCCGACGTGAATC	qRT_PCR
qAT-Val-F	GGTTTCGTGGTGTAGTTGG	qRT_PCR
qAT-Val-R	GTTCGAACCGGAGACCTTC	qRT_PCR
qAT-5s-F	GGATGCGATCATACCAGC	qRT_PCR
qAT-5s-R	GAGGGATGCAACACGAGG	qRT_PCR
qAT-Gln-F	GGTTCTATGGTGTAGTGGTTAGC	qRT_PCR
qAT-Gln-R	TACCGGGAGTCGAACCCAG	qRT_PCR
qAT-Gly-F	GCACCAGTGGTCTAGTGGTA	qRT_PCR
qAT-Gly-R	TGCACCAGCCGGGAATCGAA	qRT_PCR
qAT-Leu-F	TGTCAGAAGTGGGGTTTGAACC	qRT_PCR
qAT-Leu-R	TCAGGATGGCCGAGTGGTCTAA	qRT_PCR
qAT-actin	CTTTATGCCAGTGGTCGTAC	qRT_PCR
qAT-actin	CGTAGGATAGCATGTGGAAG	qRT_PCR
AD.ATRPC1_F	GTGAATTCCACCCGGGTGGGCATCGATTCAAGAGATGATTGGATCAG	Y2H
AD.ATRPC2_F	GTGAATTCCACCCGGGTGGGCATCGATTCAAGCCTCTGTGAGTTTCA	Y2H
AD.ATRPC3_F	GTGAATTCCACCCGGGTGGGCATCGATTTAAAAGTCATGAAAAAGCA	Y2H
AD.ATRPC4_1_F	GTGAATTCCACCCGGGTGGGCATCGATTCATATATTTTCGATATCTT	Y2H
AD.ATRPC4_2_F	GTGAATTCCACCCGGGTGGGCATCGATTCACTTCTGTGTTTCCATGT	Y2H
AD.ATRPC5_F	GTGAATTCCACCCGGGTGGGCATCGATTCATCGAGCTTTCTGTAAAA	Y2H
AD.ATRPC6_F	GTGAATTCCACCCGGGTGGGCATCGATCTAAAAGTCTAACCATTTCT	Y2H
AD.ATRPC7_1_F	GTGAATTCCACCCGGGTGGGCATCGATTTAATACACCTCTTCAACTA	Y2H
AD.ATRPC7_2_F	GTGAATTCCACCCGGGTGGGCATCGATTTAATACACCTCTTCAAATC	Y2H
AD.ATRPC8_F	GTGAATTCCACCCGGGTGGGCATCGATTCACTCTTCTTGATCAACCT	Y2H
AD.ATRPC9_1_F	GTGAATTCCACCCGGGTGGGCATCGATTCACGACTGTTCACCATTCT	Y2H
AD.ATRPC9_2_F	GTGAATTCCACCCGGGTGGGCATCGATTCACGACTGTTCACCATTCT	Y2H

AD.ATRPC10_1_F	GTGAATTCCACCCGGGTGGGCATCGATTCATTCCTCACGCCAAGTGA	Y2H
AD.ATRPC10_2_F	GTGAATTCCACCCGGGTGGGCATCGATTCATTCCTCACGCCAAGTGA	Y2H
AD.ATRPAC1_F	GTGAATTCCACCCGGGTGGGCATCGATTCAGGAGAGTTCAGAGATTA	Y2H
AD.ATRPAC2_F	GTGAATTCCACCCGGGTGGGCATCGATTCAGTTGTTTTCAATGTCCA	Y2H
AD.ATRPABC1_F	GTGAATTCCACCCGGGTGGGCATCGATTTATACAACATAACGATAGG	Y2H
AD.ATRPABC2_1_F	GTGAATTCCACCCGGGTGGGCATCGATTCAATCACCACCAACTTGAC	Y2H
AD.ATRPABC2_2_F	GTGAATTCCACCCGGGTGGGCATCGATTCAATCACCACCGACTTGAC	Y2H
AD.ATRPABC3_1_F	GTGAATTCCACCCGGGTGGGCATCGATTCACAGCTTCCTCATGAGTA	Y2H
AD.ATRPABC3_2_F	GTGAATTCCACCCGGGTGGGCATCGATTCAAAGCTTCCTCATGAGTA	Y2H
AD.ATRPABC4_F	GTGAATTCCACCCGGGTGGGCATCGATTCAGCGAGCTTCGTATTGAA	Y2H
AD.ATRPABC5_F	GTGAATTCCACCCGGGTGGGCATCGATCTAACTGTTGTCTGATTTCT	Y2H
AD.ATRPC1_R	CTCGAGCTCGATGGATCCCGTATCGATATGGAGACGAAGATGGAAAT	Y2H
AD.ATRPC2_R	CTCGAGCTCGATGGATCCCGTATCGATATGGGACTTGATCAAGAAGA	Y2H
AD.ATRPC3_R	CTCGAGCTCGATGGATCCCGTATCGATATGTCTATGTCTGAGTTTGG	Y2H
AD.ATRPC4_1_R	CTCGAGCTCGATGGATCCCGTATCGATATGTATCATACAGTTCCTGC	Y2H
AD.ATRPC4_2_R	CTCGAGCTCGATGGATCCCGTATCGATATGGATTCAGGCGAACAAAA	Y2H
AD.ATRPC5_R	CTCGAGCTCGATGGATCCCGTATCGATATGGACTTTGATGATGATGA	Y2H
AD.ATRPC6_R	CTCGAGCTCGATGGATCCCGTATCGATATGAGTAAACGAAAACGTCC	Y2H
AD.ATRPC7_1_R	CTCGAGCTCGATGGATCCCGTATCGATATGTCTTGGAAAGGAGCCCG	Y2H
AD.ATRPC7_2_R	CTCGAGCTCGATGGATCCCGTATCGATATGTCTTGGAAAGGAGGCCG	Y2H
AD.ATRPC8_R	CTCGAGCTCGATGGATCCCGTATCGATATGTTTTATCTTAGCGAGCT	Y2H
AD.ATRPC9_1_R	CTCGAGCTCGATGGATCCCGTATCGATATGAAAATAGTCAAGGCAAA	Y2H
AD.ATRPC9_2_R	CTCGAGCTCGATGGATCCCGTATCGATATGAAGGCAAATGCAGGAGC	Y2H
AD.ATRPC10_1_R	CTCGAGCTCGATGGATCCCGTATCGATATGGAGTTTTGTCCAACATG	Y2H
AD.ATRPC10_2_R	CTCGAGCTCGATGGATCCCGTATCGATATGGAGTTTTGTCCCACATG	Y2H
AD.ATRPAC1_R	CTCGAGCTCGATGGATCCCGTATCGATATGGGGACTAACGAAGTAAC	Y2H
AD.ATRPAC2_R	CTCGAGCTCGATGGATCCCGTATCGATATGGAGCACGGTTCGTTCAC	Y2H
AD.ATRPABC1_R	CTCGAGCTCGATGGATCCCGTATCGATATGTTGACGGAAGAGGAGCT	Y2H
AD.ATRPABC2_1_R	CTCGAGCTCGATGGATCCCGTATCGATATGGCTGACGAAGATTACAA	Y2H
AD.ATRPABC2_2_R	CTCGAGCTCGATGGATCCCGTATCGATATGGCTGACGACGATTACAA	Y2H
AD.ATRPABC3_1_R	CTCGAGCTCGATGGATCCCGTATCGATATGGCGAGCAATATCATCTT	Y2H
AD.ATRPABC3_2_R	CTCGAGCTCGATGGATCCCGTATCGATATGGCGAGCAATATTATCAT	Y2H
AD.ATRPABC4_R	CTCGAGCTCGATGGATCCCGTATCGATATGGATCCAGCGCCCGAACC	Y2H
AD.ATRPABC5_R	CTCGAGCTCGATGGATCCCGTATCGATATGATCATCCCTGTTCGTTG	Y2H
BD.ATRPC1_F	ATGGCCATGGAGGCCGAATTCCCGGGTCAAGAGATGATTGGATCAG	Y2H
BD.ATRPC2_F	ATGGCCATGGAGGCCGAATTCCCGGGTCAAGCCTCTGTGAGTTTCA	Y2H
BD.ATRPC3_F	ATGGCCATGGAGGCCGAATTCCCGGGTTAAAAGTCATGAAAAAGCA	Y2H
BD.ATRPC4_1_F	ATGGCCATGGAGGCCGAATTCCCGGGTCATATATTTTCGATATCTT	Y2H
BD.ATRPC4_2_F	ATGGCCATGGAGGCCGAATTCCCGGGTCACTTCTGTGTTTCCATGT	Y2H
BD.ATRPC5_F	ATGGCCATGGAGGCCGAATTCCCGGGTCATCGAGCTTTCTGTAAAA	Y2H
BD.ATRPC6_F	ATGGCCATGGAGGCCGAATTCCCGGGCTAAAAGTCTAACCATTTCT	Y2H
BD.ATRPC7_1_F	ATGGCCATGGAGGCCGAATTCCCGGGTTAATACACCTCTTCAACTA	Y2H
BD.ATRPC7_2_F	ATGGCCATGGAGGCCGAATTCCCGGGTTAATACACCTCTTCAAATC	Y2H
BD.ATRPC8_F	ATGGCCATGGAGGCCGAATTCCCGGGTCACTCTTCTTGATCAACCT	Y2H
BD.ATRPC9_1_F	ATGGCCATGGAGGCCGAATTCCCGGGTCACGACTGTTCACCATTCT	Y2H

BD.ATRPC9_2_F	ATGGCCATGGAGGCCGAATTCCCGGGTCACGACTGTTCACCATTCT	Y2H
BD.ATRPC10_1_F	ATGGCCATGGAGGCCGAATTCCCGGGTCATTCCTCACGCCAAGTGA	Y2H
BD.ATRPC10_2_F	ATGGCCATGGAGGCCGAATTCCCGGGTCATTCCTCACGCCAAGTGA	Y2H
BD.ATRPAC1_F	ATGGCCATGGAGGCCGAATTCCCGGGTCAGGAGAGTTCAGAGATTA	Y2H
BD.ATRPAC2_F	ATGGCCATGGAGGCCGAATTCCCGGGTCAGTTGTTTTCAATGTCCA	Y2H
BD.ATRPABC1_F	ATGGCCATGGAGGCCGAATTCCCGGGTTATACAACATAACGATAGG	Y2H
BD.ATRPABC2_1_F	ATGGCCATGGAGGCCGAATTCCCGGGTCAATCACCACCAACTTGAC	Y2H
BD.ATRPABC2_2_F	ATGGCCATGGAGGCCGAATTCCCGGGTCAATCACCACCGACTTGAC	Y2H
BD.ATRPABC3_1_F	ATGGCCATGGAGGCCGAATTCCCGGGTCACAGCTTCCTCATGAGTA	Y2H
BD.ATRPABC3_2_F	ATGGCCATGGAGGCCGAATTCCCGGGTCAAAGCTTCCTCATGAGTA	Y2H
BD.ATRPABC4_F	ATGGCCATGGAGGCCGAATTCCCGGGTCAGCGAGCTTCGTATTGAA	Y2H
BD.ATRPABC5_F	ATGGCCATGGAGGCCGAATTCCCGGGCTAACTGTTGTCTGATTTCT	Y2H
BD.ATRPC1_R	CCGCTGCAGGTCGACGGATCCCCGGGATGGAGACGAAGATGGAAAT	Y2H
BD.ATRPC2_R	CCGCTGCAGGTCGACGGATCCCCGGGATGGGACTTGATCAAGAAGA	Y2H
BD.ATRPC3_R	CCGCTGCAGGTCGACGGATCCCCGGGATGTCTATGTCTGAGTTTGG	Y2H
BD.ATRPC4_1_R	CCGCTGCAGGTCGACGGATCCCCGGGATGTATCATACAGTTCCTGC	Y2H
BD.ATRPC4_2_R	CCGCTGCAGGTCGACGGATCCCCGGGATGGATTCAGGCGAACAAAA	Y2H
BD.ATRPC5_R	CCGCTGCAGGTCGACGGATCCCCGGGATGGACTTTGATGATGATGA	Y2H
BD.ATRPC6_R	CCGCTGCAGGTCGACGGATCCCCGGGATGAGTAAACGAAAACGTCC	Y2H
BD.ATRPC7_1_R	CCGCTGCAGGTCGACGGATCCCCGGGATGTCTTGGAAAGGAGCCCG	Y2H
BD.ATRPC7_2_R	CCGCTGCAGGTCGACGGATCCCCGGGATGTCTTGGAAAGGAGGCCG	Y2H
BD.ATRPC8_R	CCGCTGCAGGTCGACGGATCCCCGGGATGTTTTATCTTAGCGAGCT	Y2H
BD.ATRPC9_1_R	CCGCTGCAGGTCGACGGATCCCCGGGATGAAAATAGTCAAGGCAAA	Y2H
BD.ATRPC9_2_R	CCGCTGCAGGTCGACGGATCCCCGGGATGAAGGCAAATGCAGGAGC	Y2H
BD.ATRPC10_1_R	CCGCTGCAGGTCGACGGATCCCCGGGATGGAGTTTTGTCCAACATG	Y2H
BD.ATRPC10_2_R	CCGCTGCAGGTCGACGGATCCCCGGGATGGAGTTTTGTCCCACATG	Y2H
BD.ATRPAC1_R	CCGCTGCAGGTCGACGGATCCCCGGGATGGGGACTAACGAAGTAAC	Y2H
BD.ATRPAC2_R	CCGCTGCAGGTCGACGGATCCCCGGGATGGAGCACGGTTCGTTCAC	Y2H
BD.ATRPABC1_R	CCGCTGCAGGTCGACGGATCCCCGGGATGTTGACGGAAGAGGAGCT	Y2H
BD.ATRPABC2_1_R	CCGCTGCAGGTCGACGGATCCCCGGGATGGCTGACGAAGATTACAA	Y2H
BD.ATRPABC2_2_R	CCGCTGCAGGTCGACGGATCCCCGGGATGGCTGACGACGATTACAA	Y2H
BD.ATRPABC3_1_R	CCGCTGCAGGTCGACGGATCCCCGGGATGGCGAGCAATATCATCTT	Y2H
BD.ATRPABC3_2_R	CCGCTGCAGGTCGACGGATCCCCGGGATGGCGAGCAATATTATCAT	Y2H
BD.ATRPABC4_R	CCGCTGCAGGTCGACGGATCCCCGGGATGGATCCAGCGCCCGAACC	Y2H
BD.ATRPABC5_R	CCGCTGCAGGTCGACGGATCCCCGGGATGATCATCCCTGTTCGTTG	Y2H
ATRPC1_F	CAGGTCGACTCTAGAGGATCCCCGGGTCAAGAGATGATTGGATCAG	antisense RNA
ATRPC2_F	CAGGTCGACTCTAGAGGATCCCCGGGTCAAGCCTCTGTGAGTTTCA	antisense RNA
ATRPC3_F	CAGGTCGACTCTAGAGGATCCCCGGGTTAAAAGTCATGAAAAAGCA	antisense RNA
ATRPC4_1_F	CAGGTCGACTCTAGAGGATCCCCGGGTCATATATTTTCGATATCTT	antisense RNA
ATRPC4_2_F	CAGGTCGACTCTAGAGGATCCCCGGGTCACTTCTGTGTTTCCATGT	antisense RNA
ATRPC5_F	CAGGTCGACTCTAGAGGATCCCCGGGTCATCGAGCTTTCTGTAAAA	antisense RNA
ATRPC6_F	CAGGTCGACTCTAGAGGATCCCCGGGCTAAAAGTCTAACCATTTCT	antisense RNA
ATRPC7_1_F	CAGGTCGACTCTAGAGGATCCCCGGGTTAATACACCTCTTCAACTA	antisense RNA
ATRPC7_2_F	CAGGTCGACTCTAGAGGATCCCCGGGTTAATACACCTCTTCAAATC	antisense RNA
ATRPC8_F	CAGGTCGACTCTAGAGGATCCCCGGGTCACTCTTCTTGATCAACCT	antisense RNA

ATRPC9_1_F	CAGGTCGACTCTAGAGGATCCCCGGGTCACGACTGTTCACCATTCT	antisense RNA
ATRPC9_2_F	CAGGTCGACTCTAGAGGATCCCCGGGTCACGACTGTTCACCATTCT	antisense RNA
ATRPC10_1_F	CAGGTCGACTCTAGAGGATCCCCGGGTCATTCCTCACGCCAAGTGA	antisense RNA
ATRPC10_2_F	CAGGTCGACTCTAGAGGATCCCCGGGTCATTCCTCACGCCAAGTGA	antisense RNA
ATRPAC1_F	CAGGTCGACTCTAGAGGATCCCCGGGTCAGGAGAGTTCAGAGATTA	antisense RNA
ATRPAC2_F	CAGGTCGACTCTAGAGGATCCCCGGGTCAGTTGTTTTCAATGTCCA	antisense RNA
ATRPABC1_F	CAGGTCGACTCTAGAGGATCCCCGGGTTATACAACATAACGATAGG	antisense RNA
ATRPABC2_1_F	CAGGTCGACTCTAGAGGATCCCCGGGTCAATCACCACCAACTTGAC	antisense RNA
ATRPABC2_2_F	CAGGTCGACTCTAGAGGATCCCCGGGTCAATCACCACCGACTTGAC	antisense RNA
ATRPABC3_1_F	CAGGTCGACTCTAGAGGATCCCCGGGTCACAGCTTCCTCATGAGTA	antisense RNA
ATRPABC3_2_F	CAGGTCGACTCTAGAGGATCCCCGGGTCAAAGCTTCCTCATGAGTA	antisense RNA
ATRPABC4_F	CAGGTCGACTCTAGAGGATCCCCGGGTCAGCGAGCTTCGTATTGAA	antisense RNA
ATRPABC5_F	CAGGTCGACTCTAGAGGATCCCCGGGCTAACTGTTGTCTGATTTCT	antisense RNA
ATRPC1_R	GGGGAAATTCGAGCTCGGTACCCGGGATGGAGACGAAGATGGAAAT	antisense RNA
ATRPC2_R	GGGGAAATTCGAGCTCGGTACCCGGGATGGGACTTGATCAAGAAGA	antisense RNA
ATRPC3_R	GGGGAAATTCGAGCTCGGTACCCGGGATGTCTATGTCTGAGTTTGG	antisense RNA
ATRPC4_1_R	GGGGAAATTCGAGCTCGGTACCCGGGATGTATCATACAGTTCCTGC	antisense RNA
ATRPC4_2_R	GGGGAAATTCGAGCTCGGTACCCGGGATGGATTCAGGCGAACAAAA	antisense RNA
ATRPC5_R	GGGGAAATTCGAGCTCGGTACCCGGGATGGACTTTGATGATGATGA	antisense RNA
ATRPC6_R	GGGGAAATTCGAGCTCGGTACCCGGGATGAGTAAACGAAAACGTCC	antisense RNA
ATRPC7_1_R	GGGGAAATTCGAGCTCGGTACCCGGGATGTCTTGGAAAGGAGCCCG	antisense RNA
ATRPC7_2_R	GGGGAAATTCGAGCTCGGTACCCGGGATGTCTTGGAAAGGAGGCCG	antisense RNA
ATRPC8_R	GGGGAAATTCGAGCTCGGTACCCGGGATGTTTTATCTTAGCGAGCT	antisense RNA
ATRPC9_1_R	GGGGAAATTCGAGCTCGGTACCCGGGATGAAAATAGTCAAGGCAAA	antisense RNA
ATRPC9_2_R	GGGGAAATTCGAGCTCGGTACCCGGGATGAAGGCAAATGCAGGAGC	antisense RNA
ATRPC10_1_R	GGGGAAATTCGAGCTCGGTACCCGGGATGGAGTTTTGTCCAACATG	antisense RNA
ATRPC10_2_R	GGGGAAATTCGAGCTCGGTACCCGGGATGGAGTTTTGTCCCACATG	antisense RNA
ATRPAC1_R	GGGGAAATTCGAGCTCGGTACCCGGGATGGGGACTAACGAAGTAAC	antisense RNA
ATRPAC2_R	GGGGAAATTCGAGCTCGGTACCCGGGATGGAGCACGGTTCGTTCAC	antisense RNA
ATRPABC1_R	GGGGAAATTCGAGCTCGGTACCCGGGATGTTGACGGAAGAGGAGCT	antisense RNA
ATRPABC2_1_R	GGGGAAATTCGAGCTCGGTACCCGGGATGGCTGACGAAGATTACAA	antisense RNA
ATRPABC2_2_R	GGGGAAATTCGAGCTCGGTACCCGGGATGGCTGACGACGATTACAA	antisense RNA
ATRPABC3_1_R	GGGGAAATTCGAGCTCGGTACCCGGGATGGCGAGCAATATCATCTT	antisense RNA
ATRPABC3_2_R	GGGGAAATTCGAGCTCGGTACCCGGGATGGCGAGCAATATTATCAT	antisense RNA
ATRPABC4_R	GGGGAAATTCGAGCTCGGTACCCGGGATGGATCCAGCGCCCGAACC	antisense RNA
ATRPABC5_R	GGGGAAATTCGAGCTCGGTACCCGGGATGATCATCCCTGTTCGTTG	antisense RNA

Supplemental Table2. Raw data quality control for RNA-seq.

Sample		Library Type	Reads Len.(bp)	Clean Reads	Clean Data(bp)	Q20(%)	Q30(%)	GC(%)
WT-1	R1	Paired-End	150	19,834,816	2,975,222,400	99.97%	98.48%	46
WT-1	R2	Paired-End	150	19,834,816	2,975,222,400	99.97%	98.51%	46

WT-2	R1	Paired-End	150	23,838,015	3,575,702,250	99.97%	98.22%	46
WT-2	R2	Paired-End	150	23,838,015	3,575,702,250	99.97%	98.06%	46
WT-3	R1	Paired-End	150	22,503,155	3,375,473,250	99.97%	98.31%	46
WT-3	R2	Paired-End	150	22,503,155	3,375,473,250	99.97%	98.39%	46
rpc2-1	R1	Paired-End	150	26,274,208	3,941,131,200	99.98%	98.54%	46
rpc2-1	R2	Paired-End	150	26,274,208	3,941,131,200	99.97%	98.28%	46
rpc2-2	R1	Paired-End	150	24,974,293	3,746,143,950	99.98%	98.48%	45
rpc2-2	R2	Paired-End	150	24,974,293	3,746,143,950	99.98%	98.16%	45
rpc2-3	R1	Paired-End	150	22,931,583	3,439,737,450	99.98%	98.59%	46
rpc2-3	R2	Paired-End	150	22,931,583	3,439,737,450	99.98%	98.50%	46
rpc3-1	R1	Paired-End	150	23,780,998	3,567,149,700	99.97%	98.40%	45
rpc3-1	R2	Paired-End	150	23,780,998	3,567,149,700	99.97%	98.55%	45
rpc3-2	R1	Paired-End	150	23,901,665	3,585,249,750	99.97%	97.81%	45
rpc3-2	R2	Paired-End	150	23,901,665	3,585,249,750	99.97%	98.19%	45
rpc3-3	R1	Paired-End	150	21,072,480	3,160,872,000	99.97%	98.52%	45
rpc3-3	R2	Paired-End	150	21,072,480	3,160,872,000	99.97%	98.46%	45
rpc8-1	R1	Paired-End	150	23,827,080	3,574,062,000	99.98%	98.37%	45
rpc8-1	R2	Paired-End	150	23,827,080	3,574,062,000	99.98%	98.32%	45
rpc8-2	R1	Paired-End	150	23,257,743	3,488,661,450	99.99%	98.52%	45
rpc8-2	R2	Paired-End	150	23,257,743	3,488,661,450	99.99%	98.28%	45
rpc8-3	R1	Paired-End	150	19,549,636	2,932,445,400	100.00%	98.62%	45
rpc8-3	R2	Paired-End	150	19,549,636	2,932,445,400	99.99%	98.72%	45
rpabc1-1	R1	Paired-End	150	25,738,293	3,860,743,950	99.98%	98.59%	46
rpabc1-1	R2	Paired-End	150	25,738,293	3,860,743,950	99.98%	98.76%	46
rpabc1-2	R1	Paired-End	150	24,249,992	3,637,498,800	99.98%	98.31%	46
rpabc1-2	R2	Paired-End	150	24,249,992	3,637,498,800	99.97%	98.26%	46
rpabc1-3	R1	Paired-End	150	22,128,255	3,319,238,250	99.98%	98.36%	46
rpabc1-3	R2	Paired-End	150	22,128,255	3,319,238,250	99.97%	98.11%	46
rpabc2-1	R1	Paired-End	150	22,768,031	3,415,204,650	99.98%	98.30%	46
rpabc2-1	R2	Paired-End	150	22,768,031	3,415,204,650	99.98%	98.25%	46
rpabc2-2	R1	Paired-End	150	21,484,660	3,222,699,000	99.99%	98.64%	45
rpabc2-2	R2	Paired-End	150	21,484,660	3,222,699,000	99.99%	98.79%	45
rpabc2-3	R1	Paired-End	150	23,153,055	3,472,958,250	99.99%	98.61%	46
rpabc2-3	R2	Paired-End	150	23,153,055	3,472,958,250	99.99%	98.52%	46

Supplemental Table3. Mapped data quality summary for RNA-seq.

	Total (Tags)	CDS_ Exons	%	Introns (Tags)	%	5'UTR_ Exons	%	3'UTR_ Exons	%

		(Tags)				(Tags)		(Tags)	
WT-1	31,874,446	28,809,857	90.39	135,284	0.42	541,293	1.84	2,388,012	7.52
WT-2	41,964,479	38,236,859	91.12	261,270	0.62	882,268	2.24	2,584,082	6.20
WT-3	40,841,539	37,017,709	90.64	193,875	0.47	763,981	2.01	2,865,974	7.05
nrpc2-1	36,849,447	33,015,423	89.60	193,397	0.52	596,063	1.76	3,044,564	8.31
nrpc2-2	40,858,988	36,630,778	89.65	210,565	0.52	720,658	1.92	3,296,987	8.11
nrpc2-3	35,523,106	31,869,290	89.71	150,784	0.42	617,872	1.89	2,885,160	8.16
nrpc3-1	39,117,524	34,742,571	88.82	186,386	0.48	686,395	1.93	3,502,172	9.00
nrpc3-2	37,344,773	32,971,239	88.29	177,584	0.48	549,403	1.63	3,646,547	9.81
nrpc3-3	36,288,917	31,994,235	88.17	158,098	0.44	547,144	1.67	3,589,440	9.93
nrpc8-1	35,806,247	31,329,240	87.50	286,294	0.80	480,258	1.50	3,710,455	10.45
nrpc8-2	38,047,933	34,201,304	89.89	213,788	0.56	667,850	1.90	2,964,991	7.84
nrpc8-3	34,761,280	31,445,533	90.46	233,624	0.67	753,749	2.32	2,328,374	6.74
nrpabc1-1	37,142,842	33,208,365	89.41	192,119	0.52	620,315	1.82	3,122,043	8.45
nrpabc1-2	33,934,192	30,331,896	89.38	177,270	0.52	549,344	1.77	2,875,682	8.52
nrpabc1-3	31,427,842	28,156,065	89.59	179,752	0.57	510,882	1.77	2,581,143	8.26
nrpabc2-1	37,194,447	33,383,197	89.75	183,714	0.49	659,953	1.93	2,967,583	8.02
nrpabc2-2	38,423,503	34,619,680	90.10	195,378	0.51	760,252	2.14	2,848,193	7.45
nrpabc2-3	38,888,184	35,102,282	90.26	191,185	0.49	811,718	2.25	2,782,999	7.19
